# Totally endoscopic mitral valve surgery: early experience in 188 patients

**DOI:** 10.1186/s13019-021-01464-4

**Published:** 2021-04-17

**Authors:** Yi Chen, Ling-chen Huang, Dao-zhong Chen, Liang-wan Chen, Zi-he Zheng, Xiao-fu Dai

**Affiliations:** Department of Cardiovascular Surgery, Union Hospital, Fujian Medical University, Fuzhou, 350001 People’s Republic of China

**Keywords:** Minimally invasive, Totally endoscopic, Mitral valve surgery

## Abstract

**Introduction:**

Totally endoscopic technique has been widely used in cardiac surgery, and minimally invasive totally endoscopic mitral valve surgery has been developed as an alternative to median sternotomy for many patients with mitral valve disease. In this study, we describe our experience about a modified minimally invasive totally endoscopic mitral valve surgery and reported the preliminary results of totally endoscopic mitral valve surgery. The aim of this retrospective study is to evaluate the results of totally endoscopic technique in mitral valve surgery.

**Material and methods:**

We retrospectively reviewed the profiles of 188 patients who were treated for mitral valve disease by modified totally endoscopic mitral valve surgery at our institution between January 2019 and December 2020. The procedure was performed under endoscopic right minithoracotomy and with femoro-femoral cannulation using the single two-stage venous cannula.

**Results:**

A total of 188 patients underwent total endoscopic mitral valve surgery. Fifty-six patients had concomitant tricuspid valvuloplasty, 11 patients underwent concomitant ablation of atrial fibrillation and atrial septal defect repair was performed in three patients. Only one patient postoperatively died of multi-organ failure. Two patients were converted to median sternotomy. Except for one patient underwent operation to stop the bleeding from the incision site, no other serious complications nor reintervention occurred during the follow-up period.

**Conclusions:**

The modified totally endoscopic mitral valve surgery performed at our institution is technically feasible and safe with the same efficacy as reported studies.

## Introduction

In recent years, minimally invasive techniques have been widely used in cardiac surgery, which were used for coronary artery bypass grafting, mitral, tricuspid and aortic valve surgery, atrial septal defect repair, epicardial pacemaker implantation and ablation of atrial fibrillation. Among those, the mitral valve surgery has been the most commonly used aspect of totally endoscopic technique. Minimally invasive mitral valve surgery has developed as an alternative to median sternotomy for many patients with mitral valve disease [1]. Several studies have reported the principles and outcomes of minimally invasive mitral valve surgery [2–4]. Some studies even show better results, with similar mortality and complications, but less pain and better aesthetic outcomes, compared to median sternotomy mitral valve surgery.

The type of musculoskeletal incisions remains the focus of minimally invasive cardiac surgery. Lower hemisternotomy, direct-vision right minithoracotomy and endoscopic right minithoracotomy and other approaches have been used with increasing frequency for heart valve surgery. At our institution, we performed a modified minimally mitral valve surgery using endoscopic right minithoracotomy and established cardiopulmonary bypass (CPB) by femoro-femoral cannulation. An advantage of totally endoscopic surgery is that it does not destroy the skeletal structure, with better postoperative aesthetic outcomes and less pain. In addition to potential benefits in terms of reduced blood loss, reduced morbidity and faster recovery during hospitalization [5]. The main disadvantage is the long duration of the surgery including the CPB time.

In this study, we describe our experience about a modified totally endoscopic approach mitral valve surgery with femoro-femoral cannulation using a single two stage cannula that avoids the jugular cannulation. We also report the preliminary results of totally endoscopic mitral valve surgery. The aim of this retrospective study was to evaluate the effectiveness of the modified totally endoscopic technique in mitral valve surgery.

## Materials and methods

### Patient selection and data collection

This is a single-centre, retrospective, observational study of the clinical profiles of a total of 188 consecutive patients who underwent totally endoscopic mitral valve surgery with CPB established only through femoral artery and femoral vein cannulation in our institute from January 2019 to December 2020. The study was approved by the ethics committee of the Union Hospital. Written informed consent was waived because the study was retrospective.

The exclusion criteria for this procedure were diffuse coronary artery disease, severe peripheral vascular disease, severe aortic valve disease, extensive mitral annular calcification and severe pleural adhesions from previous right-sided thoracic surgery.

We started performing totally endoscopic mitral valve surgery in 2018 at the earliest time. The surgery performed at our institute were performed by the surgeon who had gone through the learning curve. Totally endoscopic mitral valve surgery has become the preferred method for patients with mitral valve disease at our institution.

### Surgical technique

#### Anesthesia and surgical preparation

**Fig. 1:**
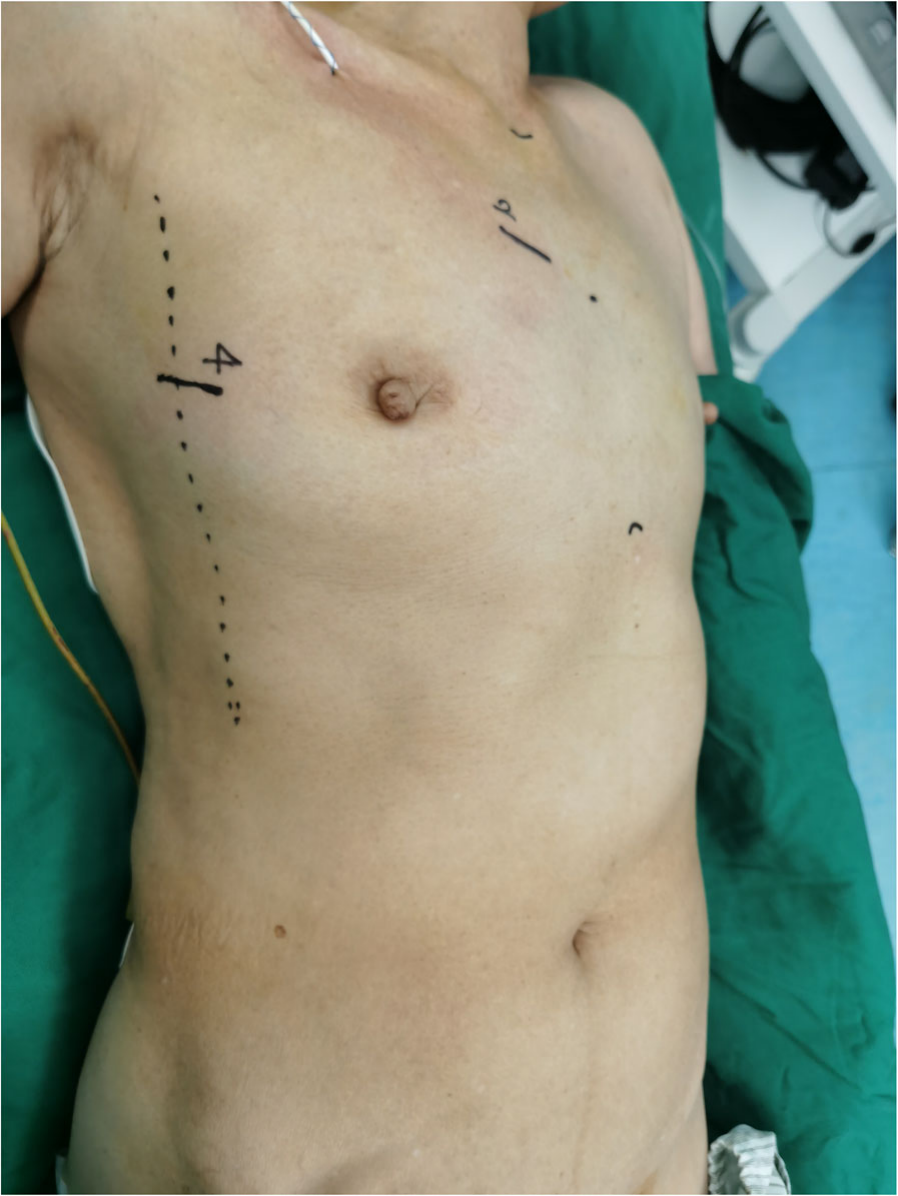
Surgical preparation and set the incision line

The procedure was performed under general anesthesia. Patients were intubated with a double-lumen endotracheal tube or a single-lumen endotracheal tube with bronchial blocker to provide for right-lung isolation. Intravenous access was obtained via the left internal jugular vein or left subclavian vein, leaving the right internal jugular vein for superior vena cava cannulation if necessary. Left brachial arterial blood pressure monitoring was utilized. The patient was placed in a supine position with a pillow under the right scapula to elevate the right hemithorax. The right elbow was slightly flexed with right shoulder abduction and then fixed the right forearm on the operating table to facilitate the exposure of the right axillary midline. Intraoperative transesophageal echocardiography (TEE) was important for intravenous cannulation positioning and accurate assessment of cardiac function, mitral valve condition and the de-airing of the heart. Two external defibrillator pads were placed in the chest wall outside the surgical field (Fig. 1).

#### Femoral vessel cannulation

CPB was utilized in all patients with femoral artery and vein cannulation. The femoral vessels were exposed through a 3–4 cm vertical incision in the groin. Two single 5.0 Prolene purse-string sutures were made on the artery and vein respectively. Femoral venous cannulation was done using the Seldinger technique under TEE guidance. A single two-stage femoral venous cannula was positioned correctly in the superior and inferior vena cava. Arterial cannulation was also performed using the Seldinger technique.

#### Surgical approach

The totally endoscopic approach was performed via an endoscopic right minithoracotomy. A 2-4 cm primary incision was usually made in the anterior axillary line in the fourth intercostal space, according to the position of the hilum on the chest film. Using the soft-tissue retractors to enhance the exposure of surgical field and protect the incision without fracturing the rib. The primary incision was for placing the 30° thoracoscope, the left ventricular venting catheter, cardioplegia needle, CO2 delivery tube, vena cava occlusion tapes and transthoracic Chitwood aortic clamp [6]. Two 2-4 cm additional working ports were installed in the secondary and fifth intercostal spaces for surgical manipulation and insertion of the valve prosthesis.

#### Surgical process

**Fig. 2:**
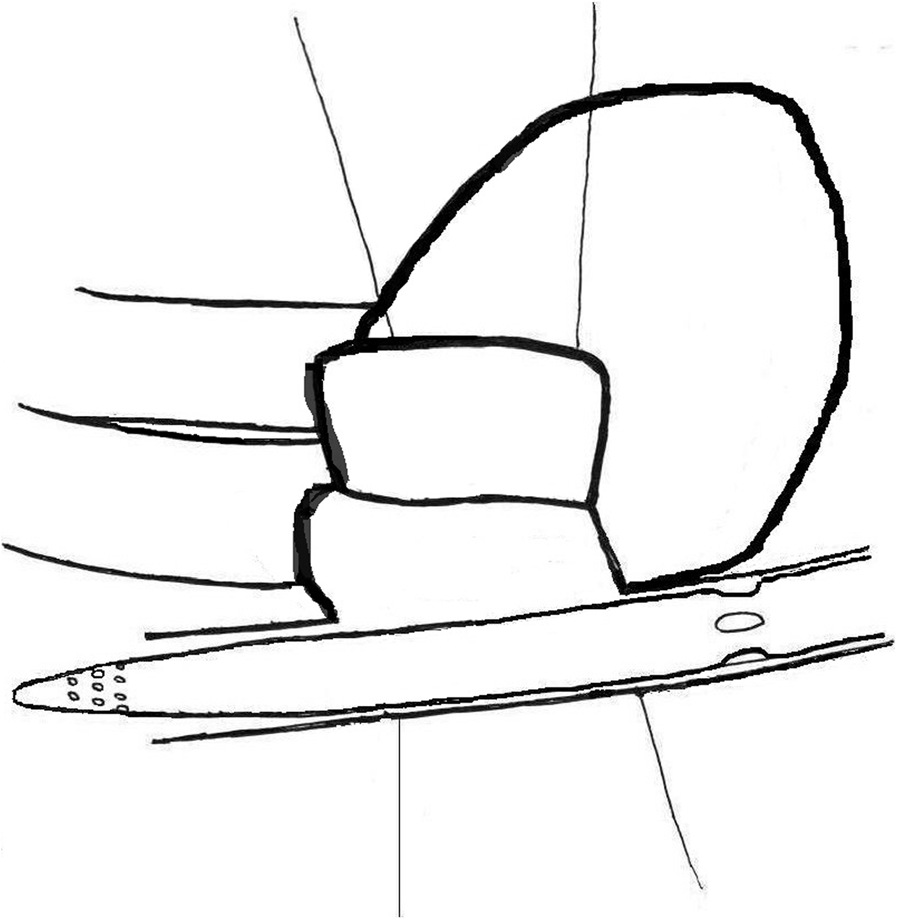
Schematic diagram of the operation field

**Fig. 3:**
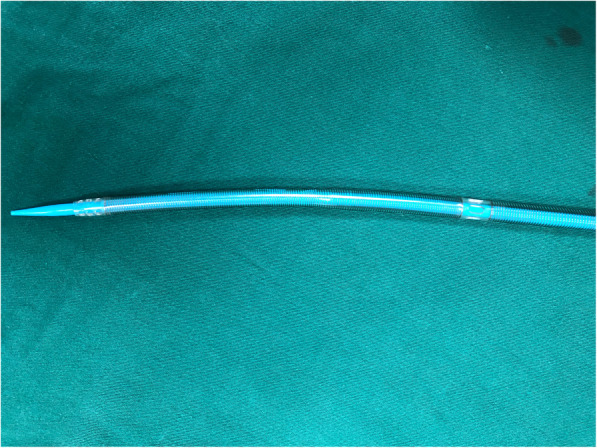
A single two-stage venous cannula

**Fig. 4:**
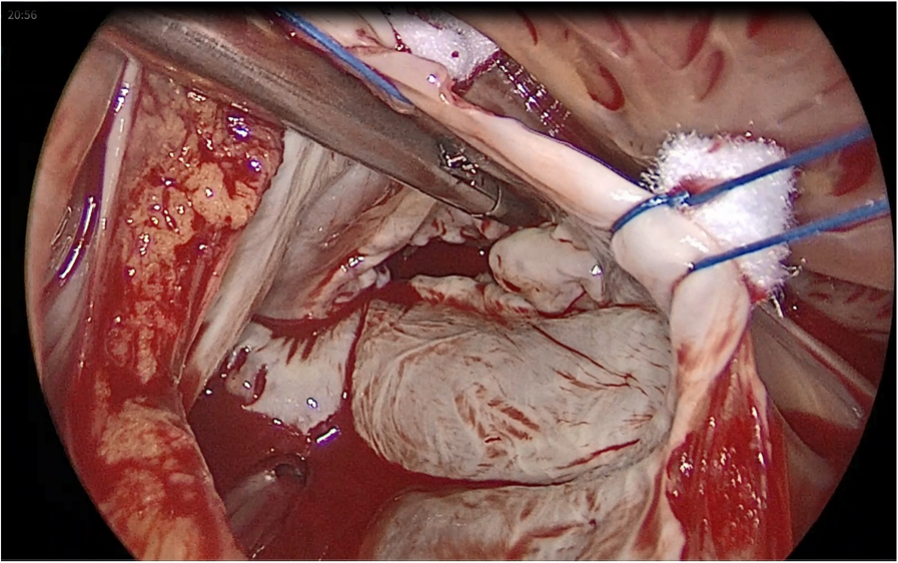
Mitral valve surgery using the totally endoscopic technique

We preferred to incise the pericardium close to the sternum to create a large flap. The pericardial margin was retracted by silk stay sutures which are then brought through the chest wall to hold the right lung back, so created a large cavity for operating. When full bypass flow and moderate hypothermia were achieved, the cava tapes were used to secure the vena cava, and the ascending aorta was occluded with Chitwood clamp. Afterwards, the antegrade HTK solution was administered and the right atrium was opened. Consecutively, the stay sutures were made on either side to retract the margin of the right atrium, which were pulled outside of the thoracic cage and were fixed on the chest wall with curved forceps. Then set the femoral venous cannula in correct position until the atrial septum can be seen (Figs. 2-3). The left atrium was entered via the atrial septum, two sets of stay sutures were used to snare the margin of the atrial septum and were pulled out of the port and secured properly (Fig. 4). After assessment, mitral valve surgery and even tricuspid valve surgery was performed. After carefully de-airing of the heart was performed and after a TEE evaluation of the heart, CPB was disconnected and all incisions were closed.

### Perioperative management

Patients were monitored in the surgical intensive care unit after surgery and transferred to the ward after hemodynamic stabilization. Routine chest X-rays and blood gas analysis were performed to rule out pulmonary complications. A transthoracic echocardiography was performed before discharge, 3 months postoperatively, and then annually after surgery to assess postoperative status.

### Statistical analysis

We define postoperative sever events according to Akin’s guideline [7]. Early mortality was defined as death within 30 days after surgery. The SPSS 24.0 was used as statistical software, and defined *P*-values < 0.05 as statistical significance. Mean ± standard deviation was calculated for quantitative data with normal distribution.

## Results

The baseline profiles of the patients are listed in Table 1. One hundred and eighty-eight patients underwent totally endoscopic mitral valve surgery (The mean age was 54.23 ± 12.81 years; 103 female). Mean BMI was 23.49 ± 1.88 kg/m^2^. Seventy-two patients were diagnosed with mitral stenosis, 78 patients with mitral regurgitation, and 38 patients with mitral stenosis and insufficiency. The mean LVED was 59.13 ± 8.24 mm. The mean LVEF was 57.79 ± 6.69%.

**Table 1:** Demographic and clinical data

Item	Data
Male/Female	85/103
Age (years)	54.23 ± 12.81
Current NYHA (median)	II
BMI (kg/m^2^)	23.49 ± 1.88
Lesion types of mitral valve	
Mitral stenosis	72
Mitral insufficiency	78
Mitral stenosis and insufficiency	38
LVED (mm)	59.13 ± 8.24
LVEF (%)	57.79 ± 6.69

Table 2 summarizes the operative data. One hundred and fifteen (61.17%) patients underwent mitral valve replacement and 73 (38.83%) patients had valve repair. Seventy-six patients underwent bioprothetic valves replacement. Associated procedures were conducted as follows: concomitant tricuspid valve plasty in 56 patients (31 with DeVega annuloplasty, 23 patients using annuloplasty rings and 2 with kay procedure) and atrial fibrillation ablation in 11 patients, atrial septal repair in three patients and two patients were conversed to median sternotomy due to severe indivisible plural adhesion. Mean time of CPB was 156.23 ± 35.17 mins, aortic cross-clamp time was 102.55 ± 17.15 min.

**Table 2:** Intra-operative data

Item	Data
Surgery strategy	
Mitral valve repair	73
Mitral valve replacement	115
Bioprothetic valves	76
Concomitant procedures	
Tricuspid valve plasty	56
Atrial fibrillation ablation	11
Atrial Septal repair	3
Convert to sternotomy	2
Cardiopulmonary bypass time (min)	156.23 ± 35.17
Aortic cross-clamping time (min)	102.55 ± 17.15

One of the patients died due to multiorgan failure (Table 3). Except for one patient underwent operation to stop the bleeding from the incision site, no other serious complications nor reintervention occurred during the follow-up period. In this study, there were two patients with bleeding events, one patient had gastrointestinal bleeding due to anticoagulation and another with bleeding from the incision site after drainage tube pulled out. Three case of inguinal lymphatic leakage and another one with right femoral vein thrombosis was reported after minimally invasive surgery. Individual six patients with pneumothorax and five patients with subcutaneous emphysema. The mean LVED was 55.72 ± 6.55 mm. The mean LVEF was 53.88 ± 8.43%. Median of NYHA was class I. The length of intensive care unit stay were 2.03 ± 1.35 days.

**Table 3:** Postoperative Data

Item	TA group
Early Mortality (<30 days)	1
Morbidity	
Structural Valve Deterioration	0
Nonstructural Dysfunction	0
Valve Thrombosis	0
Embolism	1
Bleeding Event	2
Operated Valve Endocarditis	0
Reintervention	1
Poor groin wound healing	3
Poor thoracotomy wound healing	2
Pneumothorax	6
Subcutaneous emphysema	5
LVEF (%)	53.88 ± 8.43
LVED (mm)	55.72 ± 6.55
ICU stay (days)	2.03 ± 1.35
Current NYHA (median)	I

## Discussion

Due to the rising demand for minimally invasive surgery from patients, minimally invasive mitral valve surgery techniques continue to evolve and are becoming an available alternative to median sternotomy mitral valve surgery. Since Navia and Cosgrove and Cohn first performed minimally invasive mitral valve surgery [8, 9], there have been four main minimally invasive approaches. The approaches include the following: lower hemi-sternotomy, direct-vision right minithoracotomy, endoscopic right minithoracotomy, and robotic-assisted right minithoracotomy.

Lower hemi-sternotomy is an option for minimally invasive mitral valve surgery (MIMVS) [10]. The main advantage of this approach is the ability to directly cannulate and cross-clamp the aorta. In contrast, a direct-vision right minithoracotomy approach is an attractive option for MIMVS because it causes less surgical trauma compared to sternotomy [11]. Other advantages include “straight-on” visualization of the mitral valve, decreased blood loss, lower incidence of significant wound infections, and improved cosmesis [4, 12–16]. In this way, the primary incision is placed at the level of the hilum, usually in the fourth intercostal space, starting at the midclavicular line and extending laterally to the anterior axillary line. The incision should be large enough to allow adequate light to reach the mitral valve. A head lamp may be useful with this technique. A total of approximately 6.0 cm of anterior lateral sub-mammary skin incision is required, and a rib retractor is required at the fourth intercostal space, which can cause severe postoperative pain and may result in rib fracture or intercostal vascular/neural injury. And with this approach, the visualization and lighting of subvalvular apparatus is often cumbersome.

To avoid these potential risks, totally endoscopic surgery can be performed using a 2D thoracoscope without rib spread. Minimally invasive totally endoscopic mitral valve surgery has become increasingly accepted as the norm. The totally endoscopic surgery we perform represents the smallest surgical access available, with no disruption to the sternum and ribs, limited only by the size of the prosthesis and soft tissue retractor. Establishment of extracorporeal circulation only through the femoral artery and vein with a single two-stage femoral venous cannula and an artery cannula. Perform an aesthetically pleasing right minithoracotomy. A lengthened cardioplegia perfusion cannula and transthoracic aortic clamp were introduced.

We have made appropriate improvements over the currently widely used minimally invasive procedures. The use of the single two-stage cannula has many advantages. It avoids the need for insertion of a right internal jugular vein cannula, which can lead to complications such as bleeding, hematoma, carotid artery injury and pneumothorax. In addition, the time required for preoperative preparation of the patient is greatly reduced because jugular vein cannulation is avoided. Although some may argue that air may be entrained in the venous cannula when the right atrium is open, this can be safely avoided if the two perforated segments are positioned correctly. Another issue may be due to the non-perforated section of the cannula crossing the right atrium, which may limit exposure of the tricuspid valve. However, as shown in Fig. 2, the non-perforated segment of the cannula is located at the septum and does not obstruct the valve view. In conclusion, the single two stage cannula can be safely used during surgery in the right atrium, and it allows the pump to function properly, either when the left atrium is retracted during mitral valve surgery or when the right atrium is opened during tricuspid valve surgery. In fact, it represents our preferred method of venous return during totally endoscopic surgery.

This approach provides the highest level of cosmetic results and minimizes discomfort. Casselman et al. reported that 93.5% of patients with non-rib-spreading surgery experienced little or no surgery-related pain with excellent cosmetic results [17].

1998, with the introduction of the da Vinci Surgical System, a robotic remotely operated surgical system with 3D cameras, Carpentier performs first successful robotic heart surgery [18]. However, robotic surgery has not been widely used for various reasons. First the robotic arm still needs to make two or more ports in the chest. Secondly, the main drawback of this remotely operated system is the lack of tactile feedback. Thus, the surgeon relies solely on “visual feedback” during the procedure. In addition, the high price of robotic systems, the use of a limited number of robotic instruments, and the need for two surgeons, a console surgeon and a bedside surgeon, greatly drains medical manpower and patient financial resources.

Therefore, a totally endoscopic mitral valve surgery without robotic assistance may be a reasonable option. A meta-analysis by Modi et al. identified 10 papers published between 1998 and 2005 that were suitable for analysis. The study included 1358 minimally invasive patients and 1469 sternotomy patients. No difference in mortality, stroke, reoperation bleeding, new-onset atrial fibrillation, ICU hospitalization or length of stay despite longer cross-clamp and extracorporeal circulation times in the minimally invasive treatment group [5]. Galloway of New York University reported the longest results to date of minimally invasive mitral valve surgery. Between 1996 and 2008, they performed 1071 minimally invasive mitral valve repair and compared their results with 1601 routine procedures. They reported a perioperative mortality rate of 1.3% in both groups of patients with isolated mitral valve repair, and no difference in major adverse events. The long-term outcome is equivalent to median sternotomy [19]. As one of the consistent findings of various case series over the last decade, it is clear that MIMVS has more operative time (extracorporeal circulation and cross-clamp time) than conventional surgery. Of all the potential benefits of MIMVS, pain relief and a faster return to normal activity were the most consistent findings.

In this study, the mortality rate was 0.53%, which is close to the results reported in the appellate literature, and in addition there was no significant increase in the incidence of serious complications. Therefore, in terms of post-operative mortality and serious complications, the short-term outcomes of totally endoscopic mitral valve surgery in our center are very impressive.

Totally endoscopic surgery for mitral valve disease is technically challenging and its use is currently limited to a small number of experienced surgeons, as it requires surgeons to overcome a lengthy learning curve [20, 21]. Prior to the start of this study, the surgical group had performed multiple hemisternotomy, direct-vision right minithoracotomy cardiac surgery as well as more than 20 totally endoscopic cardiac surgeries, had already gone through the learning curve. This study is representative in the local area.

This study has some limitations owing to retrospective single-institution small-volume observational study design without a control group. Only one surgeon performed the procedures; therefore, inter-operator differences could not be studied.

## Conclusions

We have performed 188 mitral valve surgery under the modified totally endoscopic approach. Mitral valve replacement or repair was successfully performed by a team of experienced surgeons through femoral artery and vein cannulation with three 2–4 cm long chest wall incisions. Other procedures include atrial septal defect repair and atrial fibrillation ablation and tricuspid valvuloplasty. In addition to minimal invasiveness and expedited postoperative recovery, mitral valve surgery with the aid of endoscopic visual guidance and TEE monitoring is a safe and effective surgical approach. In total, totally endoscopic surgery for routine mitral disease deserves to be widely advocated. In summary, totally endoscopic surgery for mitral valve disease by an experienced operator is feasible, safe, effective, and worthy of widespread adoption.

## Data Availability

Data sharing not applicable to this article as no data sets were generated or analyzed during the current study.

## Abbreviations

TEE: Transesophageal echocardiography
CPB: Cardiopulmonary bypass
NYHA class: New York Heart Association functional classification
BMI: Body mass index
LVED: Left ventricular end diastolic
LVEF: Left ventricular ejection fraction
MIMVS: Minimally invasive mitral valve surgery
